# MR histology reveals tissue features beneath heterogeneous MRI signal in genetically engineered mouse models of sarcoma

**DOI:** 10.3389/fonc.2024.1287479

**Published:** 2024-05-31

**Authors:** Stephanie J. Blocker, Yvonne M. Mowery, Jeffrey I. Everitt, James Cook, Gary Price Cofer, Yi Qi, Alex M. Bassil, Eric S. Xu, David G. Kirsch, Cristian T. Badea, G. Allan Johnson

**Affiliations:** ^1^ Department of Radiology, Duke University Medical Center, Duke University, Durham, NC, United States; ^2^ Department of Radiation Oncology, Hillman Cancer Center, University of Pittsburgh Medical Center, Pittsburgh, PA, United States; ^3^ Department of Pathology, Duke University Medical Center, Duke University, Durham, NC, United States; ^4^ Department of Radiation Oncology, Duke University Medical Center, Duke University, Durham, NC, United States; ^5^ Duke University Medical Center, Duke University, Durham, NC, United States; ^6^ Departments of Radiation Oncology and Medical Biophysics, Princess Margaret Cancer Centre, University Health Network (UHN), Toronto, ON, Canada

**Keywords:** MRI, sarcoma, histology, image registration, multi-modal, preclinical, feature mapping

## Abstract

**Purpose:**

To identify significant relationships between quantitative cytometric tissue features and quantitative MR (qMRI) intratumorally in preclinical undifferentiated pleomorphic sarcomas (UPS).

**Materials and methods:**

In a prospective study of genetically engineered mouse models of UPS, we registered imaging libraries consisting of matched multi-contrast *in vivo* MRI, three-dimensional (3D) multi-contrast high-resolution *ex vivo* MR histology (MRH), and two-dimensional (2D) tissue slides. From digitized histology we generated quantitative cytometric feature maps from whole-slide automated nuclear segmentation. We automatically segmented intratumoral regions of distinct qMRI values and measured corresponding cytometric features. Linear regression analysis was performed to compare intratumoral qMRI and tissue cytometric features, and results were corrected for multiple comparisons. Linear correlations between qMRI and cytometric features with p values of <0.05 after correction for multiple comparisons were considered significant.

**Results:**

Three features correlated with *ex vivo* apparent diffusion coefficient (ADC), and no features correlated with *in vivo* ADC. Six features demonstrated significant linear relationships with *ex vivo* T2*, and fifteen features correlated significantly with *in vivo* T2*. In both cases, nuclear Haralick texture features were the most prevalent type of feature correlated with T2*. A small group of nuclear topology features also correlated with one or both T2* contrasts, and positive trends were seen between T2* and nuclear size metrics.

**Conclusion:**

Registered multi-parametric imaging datasets can identify quantitative tissue features which contribute to UPS MR signal. T2* may provide quantitative information about nuclear morphology and pleomorphism, adding histological insights to radiological interpretation of UPS.

## Introduction

Undifferentiated pleomorphic sarcomas (UPS) form the largest subset of the 10% to 20% of soft tissue sarcomas without a clear tissue of origin (1, 2). Although relatively rare, soft tissue tumors can be diagnostically challenging, with a large number of tumor subtypes that exhibit overlapping diagnostic features (3). The genomic underpinnings of UPS are defined by their mutational complexity, including marked aneuploidy (4, 5), rather than a set of specific alterations (2, 6). This complexity can make molecular subtyping of these tumors a challenge. Morphologically, UPS can be quite heterogeneous, with atypical ovoid or spindle-shaped cells arranged in disorganized fascicular or storiform patterns. UPS tumors characteristically harbor large variations in nuclear size and shape, and may include multinucleated cells (7). Radiographic findings are variable in UPS, but they typically present with heterogeneous intratumor signal (8). Accurate diagnosis in patients with UPS relies on adequate sampling of the lesion given the heterogeneity of these tumors (9).

Although diagnosis of UPS presents a challenge, identifying relevant features is of prognostic value (10, 11). Non-invasive magnetic resonance (MR) imaging is a staple of clinical management of soft tissue sarcomas, though its capacity for prognostication has traditionally been limited to tumor size, topological features, necrosis identification, and peritumoral enhancement (10, 12). In addition to these features, the presence of the “tail sign” on MRI is prognostic, having been associated with a higher risk of local recurrence in UPS following surgical excision (13, 14). Increased signal heterogeneity on T2-weighted (T2W) MR images is typically considered a negative prognostic factor for soft tissue sarcomas (15). More recently, studies of radiomic features of soft tissues sarcoma have demonstrated prediction of tumor grade based on MR imaging (16). However, the histological foundation for MR features in UPS has not been established. Specifically, the relationship between quantitative MR imaging (qMRI) features and the hallmark cytological morphologies of UPS remains unknown. The purpose of this study was to measure relationships between qMRI and histology in UPS that may improve prognostication and decision making for patients with this challenging disease. Specifically, we looked for correlations between tissue histological features and the parameters of apparent diffusion coefficient (ADC) and T2*, as these measurements were acquired during our co-clinical imaging trial of soft tissue sarcoma (NCI U24 CA220245) performed to mirror patient imaging in the ongoing phase II clinical trial SU2C-SARC032 (NCT03092323). Our hypothesis was that by registering MR to pathology at high resolution using MRH, we would identify histological features of UPS which significantly correlate with qMRI, specifically ADC and T2*.

## Materials and methods

### Animal models

All animal studies were reviewed and approved by the Institutional Animal Care and Use Committee. Soft tissue sarcomas (n = 8) were induced in the hind limb of genetically engineered mice through a combination of Cre recombinase (Cre) activation to delete *p53* and local injection of carcinogen, as described previously (17, 18). Briefly, adenovirus expressing Cre and 300 μg 3-methylcholanthrene (MCA; Sigma-Aldrich, Saint Louis, MO) were injected into the gastrocnemius muscle of *p53^fl/fl^* mice on a 129/SvJ background. Induction was performed in both male and female mice at 6-12 weeks of age, and tumors developed in approximately 8-12 weeks. When tumors reached 500-1,000 mm^3^, imaging studies were initiated.

### Study design

**Figure 1:**
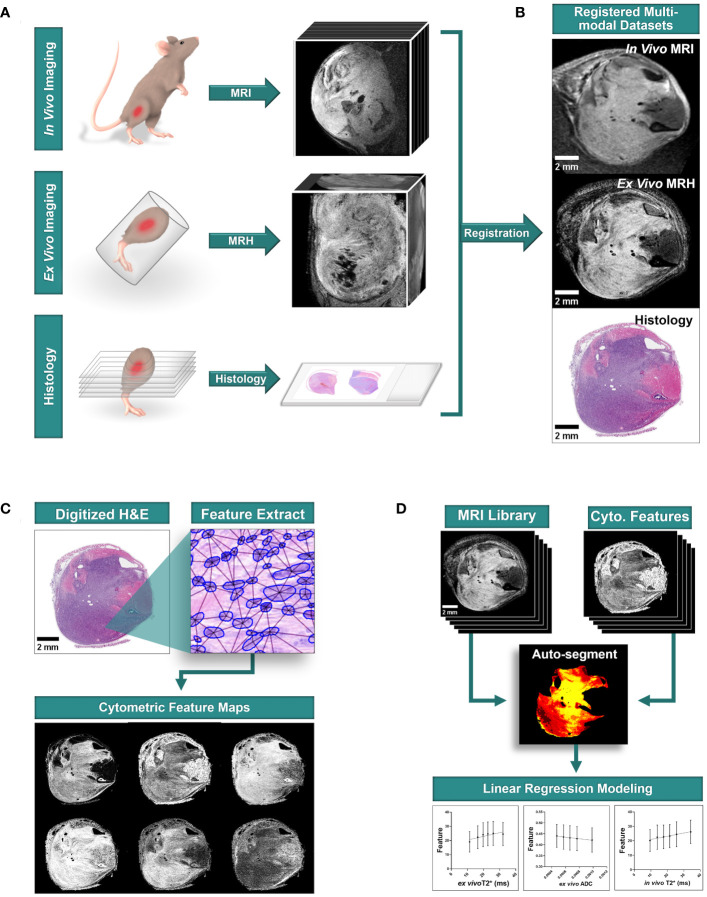
Multi-modal sarcoma imaging library registration and analysis workflow. Mouse models of UPS were imaged to construct registered imaging data libraries. **(A)** Tumors were imaged with MRI *in vivo*, and fixed tissues were imaged at high resolution with MRH before preparation for conventional histology with hematoxylin and eosin (H&E) staining. **(B)** Tumor imaging libraries were registered to histological sections. **(C)** Quantitative cytometric feature maps were generated from H&E-stained tumor cross sections. **(D)** Registered MR imaging datasets were compared to quantitative cytometrics intratumorally to probe for linear relationships between tumor features and MR signal. Scale bars = 2 mm.

This pilot study was conducted prospectively. For analysis of multi-modal imaging libraries, three-dimensional (3D) *in vivo* MRI and subsequent *ex vivo* MRH were registered together. After tissues were processed for pathology, 3D MR datasets were then registered to two-dimensional (2D) histology slides that were digitized through whole-slide imaging (**Figures 1A, B**). From digitized histology, we generated quantitative cytometric feature maps based on whole-slide automated nuclear segmentation (**Figure 1C**). In registered libraries, we automatically segmented intratumoral regions of distinct qMRI values and measured corresponding cytometric features in these regions (**Figure 1D**).

### *In vivo* MRI

*In vivo* MR images were acquired on a 7-T Biospec small-animal MRI scanner (Bruker, Billerica, MA). 2.5% atomized isoflurane in air was used to induce anesthesia, followed by maintenance at 1%-2%. Animals were imaged in the lateral recumbent position, with a four-element mouse brain coil secured over the tumor-bearing limb. The animals and surface coil were positioned on a custom 3D-printed bed with integrated tubing for circulation of warm water and equipment for active monitoring of breath rate and body temperature. Animal vitals were monitored through the duration of imaging. The unit was centered in a 72-mm diameter, actively decoupled linear volume coil for transmission. Acquisition was performed using ParaVision software, version 6.0.1 (Bruker). Images were exported in Digital Imaging and Communications in Medicine (DICOM) format. *In vivo* sequences included anatomic 2D, non-isotropic T1-weighted fast low-angle shot (FLASH), T2-weighted turbo rapid acquisition with relaxation enhancement (TurboRARE), spiral-trajectory diffusion-weighted imaging (DWI), and multigradient-recalled-echo (MGRE) sequences. Sequences were selected according to the small animal imaging protocols developed during the companion co-clinical imaging study of soft tissue sarcomas (NCI U24 CA220245), and were originally designed to mimic the clinical protocols on study. Basic scanning parameters are described in **Table 1**. Total acquisition time per animal was less than 90 minutes.

**Table 1:** Imaging Parameters for *in vivo* MR in preclinical soft tissue sarcomas.

Sequence	Parameter	Value
T1-weighted FLASH		
	TE (ms)	4.5
	TR (ms)	767.5
	Flip angle (°)	30
	No. excitations	3
	In-plane resolution (mm)	0.1 X 0.1
	Slice thickness (mm)	0.3
	Acquisition time	4.9 minutes
T2-weighted TurboRARE		
	TE (ms)	45
	TR (ms)	8000
	RARE	8
	No. of excitations	3
	In-plane resolution (mm)	0.1 X 0.1
	Slice thickness (mm)	0.3
	Acquisition time	10.7 minutes
Spiral DWI		
	*b* values (s/mm^2^)	0, 100, 300, 500
	Interleaves	40
	No. of excitations	2
	In-plane resolution (mm)	0.1 X 0.1
	Slice thickness (mm)	0.6
	Acquisition time	30 minutes
MGRE		
	TE (ms)	4, 19, 34, 49
	No. of excitations	3
	In-plane resolution (mm)	0.1 X 0.1
	Slice thickness (mm)	0.6
	Acquisition time	20 minutes

TE, echo time; TR, repetition time; FLASH, fast low-angle shot; TurboRARE, turbo rapid acquisition with relaxation enhancement; DWI, diffusion weighted imaging; MGRE, multi-gradient recalled echo.

### *Ex vivo* MR histology

Following *in vivo* imaging experiments, animals were euthanized according to Institutional Animal Care and Use Committee humane practices guidelines. Tissues were enhanced for MRH with contrast agents using previously described methods (19–21). Transcardial perfusion was performed with a 1:10 mixture of ProHance (Bracco Diagnostics, Princeton, NJ) in formalin. Fixation with a 1:10 mixture of ProHance and buffered formalin reduces the T1 by more than six-fold in most mouse tissues. This permits the use of a 3D spin echo sequence, minimizing geometric distortions with a short TR providing the signal averaging inherent in 3D spin warp encoding without excessive acquisition times. At TR=50 ms, the SNR is more than 5 times greater in the actively stained tissue than in a formalin-fixed specimen (19). T1s in the specimens in this study were typically ~ 100 ms. The resulting gain in SNR allows us to achieve spatial resolution of 35 um with scan times of ~ 8 hrs which would not be possible with unstained specimens. Tumor-bearing limbs were then resected and immersed in the ProHance/formalin and kept at 4°C. 24 hours after resection, specimens were transferred to a 1:200 mixture of ProHance in phosphate buffered saline to rehydrate tissues one week prior to *ex vivo* MRH.

Specimen imaging was performed on a 7-T horizontal bore magnet with high-performance Resonance Research gradient coils (2500 mT/m peak) controlled by an Agilent Direct Drive console (Agilent Technologies). High sensitivity was achieved using an in-house built solenoid resonator. Tissues were immersed in fomblin (Sigma-Aldrich) to reduce surface susceptibility artifacts. 3D DWI and MGRE sequences were acquired at 35 µm isotropic resolution using compressed sensing with an acceleration of 8X. DWI was performed using a 3D Stejskal-Tanner sequence. Phase-encoding was sparsely sampled in two dimensions using probabilistic methods (21–23). The data was fully sampled in the readout direction. A script running on the Agilent console automated each acquisition by launching the sequence and writing the data to a local file. At the conclusion of that acquisition, the file was automatically written to a remote disc on a high-performance Dell cluster. The script launched the reconstruction program on the cluster which started with a Fourier Transform along the fully sampled readout dimension. This produced a large collection of undersampled 2D images which were distributed to multiple nodes of the cluster for iterative reconstruction using the BART reconstruction tool box (https://mrirecon.github.io/bart/). DWI and MGRE sequences were selected to ensure that qMRI parameters (ADC and T2*) were available to complement *in vivo* qMRI measurements. DWI and MGRE sequence parameters were calibrated to ensure adequate SNR at high resolution in fixed tissues in the shortest reasonable scan time. Total acquisition time was approximately 8 hours per sample. The basic scanning parameters are included in **Table 2**.

**Table 2:** Imaging Parameters for *ex vivo* MR in preclinical soft tissue sarcomas.

Sequence	Parameter	Value
DWI		
	*b* values (s/mm^2^)	0, 500, 1000, 1500, 2000
	Compression Factor	5
	Isotropic Resolution (mm^3^)	0.035
	Acquisition time	4 hours, 33 minutes
MGRE		
	TE (ms)	6, 12, 18, 24
	Isotropic Resolution (mm^3^)	0.035
	Acquisition time	3 hours, 39 minutes

DWI, diffusion weighted imaging; MGRE, multi-gradient recalled echo; TE, echo time.

### Histological preparation

Following MRH, tumor-bearing limbs were decalcified in 14% ethylenediaminetetraacetic acid for 14 days to allow tissue preparation with intact bone. Tissues were stored in 70% ethanol, and gross sections were trimmed to tissue cassettes at 2-3 levels along the tibia/fibula prior to embedding in paraffin. 6 μm sections were cut from each level and stained with hematoxylin and eosin (H&E). Stained slides were scanned at 40X on an Aperio AT2 whole-slide scanner (Leica Biosystems, Buffalo Grove, IL), and data were stored in SVS format. The result was digitized H&E images of tumor-bearing limb cross sections at 2-3 levels for each specimen.

### Multi-modal image registration

**Figure 2:**
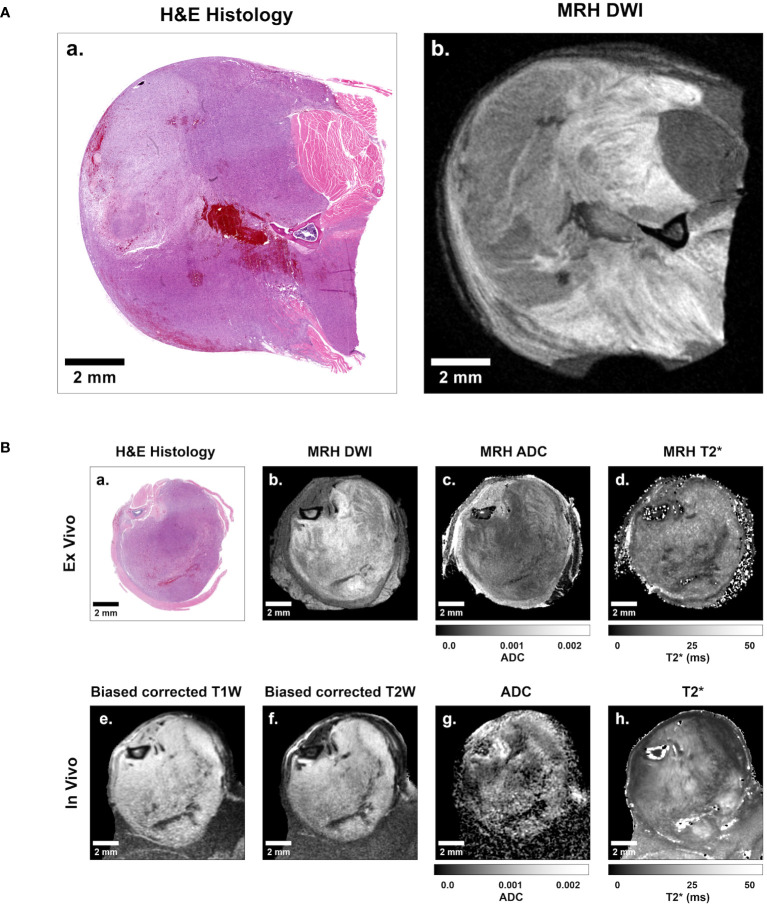
Representative sarcoma imaging library including multi-contrast, multi-resolution MR datasets registered to digitized 2D histological slides. **(A)** Shown is a sample of a digitized 2D histology image (left) with a registered 35 μm isotropic MRH diffusion-weighted image (right). **(B)** The complete imaging suite gathered for each sarcoma-bearing animal included registered *ex vivo* histology **(B.a)**, diffusion-weighted MRH **(B.b)**, MRH apparent diffusion coefficient (ADC) maps **(B.c)**, MRH T2* maps **(B.d)**, as well as *in vivo* bias corrected anatomical T1-weighted **(B.e)** and T2-weighted **(B.f)** images, ADC maps **(B.g)**, and T2* maps **(B.h)**. Scale bars = 2 mm.

For subsequent analysis of imaging libraries, 3D *in vivo* MRI and subsequent *ex vivo* MRH were registered together. After tissues were processed for pathology, 3D MR datasets were then registered to 2D digitized histology slides. Image registration was performed using 3D Slicer (https://www.slicer.org/), an open-source platform for image analysis, as described previously (18). Briefly, *in vivo* images were registered to MRH images through a series of linear (rigid) and landmarks-based (non-rigid) transformations. *Ex vivo* MRH images were registered to digitized histology via linear transformation and 2D slice selection, followed by landmarks based non-rigid transformation. The high resolution of MRH facilitates registration of multi-modal imaging datasets, as meso- and micro-scale structures are resolved. The resulting transformation was applied to all MRH-registered *in vivo* images. The result was a series of histology-registered MR images which included *in vivo* T1- and T2-weighted images, DWI, MGRE, and DTI, as well as *ex vivo* DWI and MGRE (**Figure 2**). In addition, apparent diffusion coefficient (ADC) and T2 star (T2*) qMRI maps were calculated for all DWI and MGRE image series, respectively.

Registration success was quantified by comparing tumor segmentations. Binary images defined via semi-automated tumor segmentation in MR (24) and histological images were compared post-registration transformation as described previously (18). Dice similarity coefficients (DSC) were calculated to measure registration success, with a minimum DSC cutoff of 0.8 required for study inclusion.

### Cytometric feature mapping

To generate quantitative cytometric feature maps, automated nuclear segmentation was performed on digitized H&E images using the StarDist plugin for FIJI as described previously (25, 26). 30 features broadly divided into four categories were measured for each nucleus: Topology, Delaunay triangulation distance, nuclear stain Haralick features, and stain intensity features. Nuclei were sorted into 35 μm pixels to match the registered MRH resolution, and mean and variance of features were measured for each pixel. In addition, we measured how many cells were detected in each pixel to generate density maps. The result was a library of 61 cytometric feature maps for each H&E slide, which were quantitative and spatially matched to the registered MR images.

### Image analysis

All correlative studies were performed using qMRI calculated diffusion coefficient and T2* maps derived from acquired MR data. Specifically, ADC was derived by exponential fitting of intensity across a range of *b* values, and T2* was calculated via exponential fitting of intensity from the series of echoes in MGRE. The resulting qMRI parameters are referred to as ADC and T2*, respectively, and were available for both *in vivo* and *ex vivo* data. The methods for automated segmentation and measurement of registered MR/pathology datasets were described in detail previously (18, 24). Briefly, normalized curves of ADC and T2* distribution among the entire cohort were calculated for both *in vivo* and *ex vivo* data. 6 bins were determined for each qMRI curve (ADC and T2*) with equal area under the curve, with boundaries at ± 2 standard deviations. These bins were used to automatically segment regions of differing qMRI values in histology-registered ADC and T2* images. The regions of interest were automatically measured in all 61 cytometric feature maps derived from registered histology slides. The results were cytometric feature measurements evaluated in regions of incrementally increasing ADC and T2*.

Measured features were plotted as a function of ADC or T2* for linear regression analysis. Where multiple slides were available per animal, the measured cytometric-to-MRH relationships were averaged on a per-animal basis prior to group analysis, as described previously (18, 25, 26). This ensured that the number of available slides for each subject did not impact the group analysis. Mean animal values for each cytometric feature were plotted for the cohort as a function of four qMRI parameters: *ex vivo* ADC, *ex vivo* T2*, *in vivo* ADC, and *in vivo* T2*. Tissue features which demonstrated significant non-zero relationships when plotted as a linear function of qMRI, after correction for multiple comparisons, were identified as potential contributors to qMRI in heterogeneous sarcomas.

### Statistical analysis

Statistical analyses were performed using Prism (version 9.00 for Windows; GraphPad Software, San Diego, CA). The pilot sample size was chosen based on previous MRH pilot studies, in which individual tumor specimens demonstrated significant linear relationships between measured cytometric features and qMRI (p<0.05, R^2^>0.9, Pearson’s r>0.9). Sample size (n=7) was calculated to achieve statistical power (1- β) of 0.8, when α=0.05 and *r*=0.9, with a loss of n=1 incorporated to account for any errors in registration or imaging complications. The resulting pilot study was carried out with n=8. Linear regression analysis was performed for group data comparing each cytometric feature with four qMRI parameters, and p-values describing the significance of non-zero relationships were reported, as well as R^2^ goodness of fit. For each MR sequence, correction for multiple comparisons was performed using the Benjamini-Hochberg method. Features of interest were identified as regressions with slopes that statistically deviated from zero (*P* <.05) after correction for multiple comparisons.

## Results

### Multiple tumor cytometric features correlate linearly with MR signal in a cohort of murine soft tissue sarcomas

**Figure 3:**
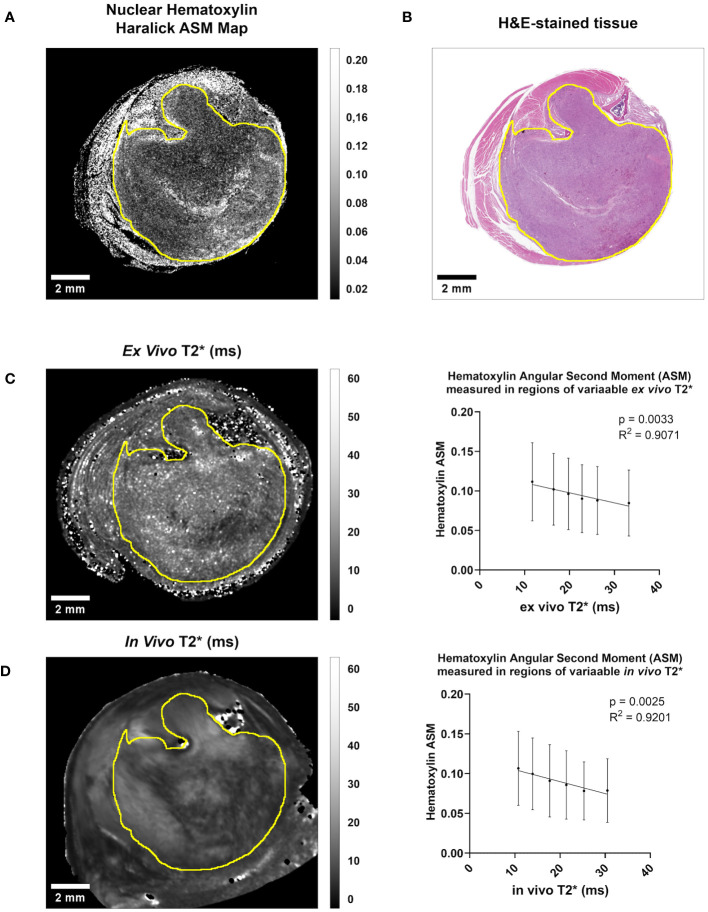
Both *in vivo* and *ex vivo* T2* demonstrate significant linear relationships with Hematoxylin Haralick Angular Second Moment (ASM) at the intratumoral level in murine soft tissue sarcomas. **(A)** A map of nuclear texture feature Haralick ASM was derived from an H&E-stained cross-section **(B)** of a soft tissue sarcoma of the hind limb. Within the tumor boundary (yellow line) Nuclear Haralick ASM demonstrated a significant linear relationship with *ex vivo* T2* in this sample **(C)**. This trend was also seen when comparing Nuclear Haralick ASM to *in vivo* T2* imaging in this sample **(D)**. Plotted are mean and SD Nuclear Haralick ASM in regions of variable T2*, and p-value and R^2^ values are included. Scale bar = 2 mm.

We successfully registered all MR images in this cohort to their corresponding histology slides, with a mean DSC of 0.944 for *ex vivo* MRH to histology, and a mean DSC of 0.930 for *in vivo* MRI to histology. Detailed metrics of registration success are provided for each specimen in **Supplementary Table 1**. Just as we have shown previously (18), we demonstrated linear relationships between intratumoral qMRI and cytometric features in individual registered datasets of preclinical soft tissue sarcomas (**Figure 3**). These relationships were often consistent in both the *ex vivo* and *in vivo* analyses for a given tissue (**Figures 3C, D**). However, due to improvements in tissue processing and histological data consistency, we were able to expand these experiments for cohort analysis (n = 8).

After correcting for multiple comparisons, we found that a subset of cytometric features correlated significantly with qMRI in the group data, including *in vivo* and *ex vivo* qMRI. Significant non-zero relationships between quantitative cytometric measurements plotted as a linear function of qMRI measurements are described in **Table 3**. Somewhat surprisingly, only three features out of 61 correlated with *ex vivo* ADC, and no features correlated with *in vivo* ADC. Complete summary tables describing the corrected p-values for all cytometric features compared to *ex vivo* and *in vivo* ADC are provided in **Supplementary Table 2** and **Supplementary Table 3**, respectively. Interestingly, six features demonstrated significant linear relationships with *ex vivo* T2*, and fifteen features correlated significantly with *in vivo* T2*. Complete summary tables describing the corrected p-values for all cytometric features compared to *ex vivo* and *in vivo* T2* are provided in **Supplementary Table 4** and **Supplementary Table 5**, respectively. In both cases, nuclear Haralick texture features were the most prevalent type of feature correlated with T2*.

**Table 3:** Cytometric features demonstrating significantly non-zero linear relationships with quantitative *in vivo* and *ex vivo* MR signal in soft tissue sarcomas (n = 8) after correction for multiple comparisons.

MR Imaging Sequence	Tissue Cytometric Feature	Linear Regression Corrected p-value	Feature Category
*Ex Vivo* ADC	Variance in Delaunay Max Distance	0.0216	Delaunay
	Variance in Delaunay Average Distance	0.0378	Delaunay
	Variance in Hematoxylin Entropy	0.0378	Nuclear Haralick
*In Vivo* ADC	N/A	–	–
*Ex Vivo* T2*	Mean Nuclear Max Diameter	0.0316	Topology
	Mean Hematoxylin ASM	<0.005	Nuclear Haralick
	Mean Hematoxylin Difference Entropy	<0.005	Nuclear Haralick
	Variance in Hematoxylin Difference Entropy	0.0065	Nuclear Haralick
	Mean Hematoxylin Sum Entropy	<0.005	Nuclear Haralick
	Variance in Hematoxylin Sum Entropy	0.0108	Nuclear Haralick
*In Vivo* T2*	Mean Nuclear Max diameter	0.0432	Topology
	Mean Nuclear Solidity	0.0412	Topology
	Variance in Delaunay Max Distance	0.0462	Delaunay
	Mean Hematoxylin ASM	<0.005	Nuclear Haralick
	Mean Hematoxylin Difference Entropy	0.0201	Nuclear Haralick
	Variance in Hematoxylin Difference Entropy	<0.005	Nuclear Haralick
	Mean Hematoxylin Entropy	<0.005	Nuclear Haralick
	Variance in Hematoxylin Entropy	0.0198	Nuclear Haralick
	Variance in Hematoxylin IDM	0.0416	Nuclear Haralick
	Variance in Hematoxylin Sum Average	0.0472	Nuclear Haralick
	Mean Hematoxylin Sum Entropy	<0.005	Nuclear Haralick
	Variance in Hematoxylin Sum Entropy	<0.005	Nuclear Haralick
	Variance in Hematoxylin Peak Intensity	0.0432	Stain
	Variance in Hematoxylin Average Intensity	0.0414	Stain
	Variance in Hematoxylin Minimum Intensity	0.0451	Stain

ADC, apparent diffusion coefficient; N/A, not applicable; ASM, angular second moment.

### T2* correlated significantly with nuclear texture and topology features in both *in vivo* MRI and *ex vivo* MRH

**Figure 4:**
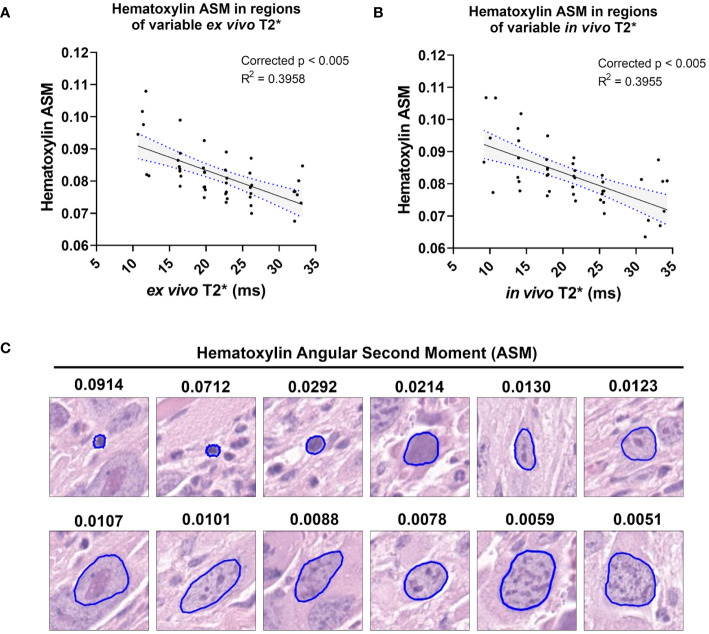
T2* correlates with a measure of nuclear stain texture, nuclear Haralick Angular Second Moment (ASM), in both *ex vivo* and *in vivo* MR. Among the cohort of soft tissue sarcomas (n = 8), regions of variable *ex vivo* T2* demonstrated a significant linear relationship with nuclear hematoxylin Haralick ASM **(A)**. The same trend was observed *in vivo* among the same cohort **(B)**. Regression lines are plotted in black, with 95% CI represented with blue dotted lines and shading. Corrected p-values and R^2^ values are provided for reference. Nuclear hematoxylin Haralick ASM is a measure of textural homogeneity, with high values corresponding to greater uniformity in intranuclear staining **(C)**. Nuclear Haralick ASM is also related to size, as shown in representative nuclei with variable ASM values. Pathology images are 25 µm^2^ tiles, with nuclear segmentations outlined in blue.

In both *ex vivo* and *in vivo* images, multiple cytometric features related to nuclear Haralick texture correlated significantly with T2*. For example, mean Haralick hematoxylin Angular Second Moment (ASM) exhibited a significant inverse linear correlation with *ex vivo* T2* (corr. p<0.005) and *in vivo* T2* (corr. p<0.005) (**Figures 4A, B**). Haralick ASM is a measure of homogeneity among grey level intensities in the measured pixels, in this case, within a nucleus stained by hematoxylin (27, 28). This is visibly evident on the pathology slide, with increased ASM demonstrating higher stain homogeneity within the nuclear boundary, and lower ASM values measured in nuclei with high stain variance, which occurs with heterogeneous chromatin structure or prominent nucleoli (**Figure 4C**). Other examples of Haralick features correlating with *ex vivo* and *in vivo* T2* include mean hematoxylin difference entropy (*ex vivo*, corr. p<0.005; *in vivo* corr. p=0.0201), variance in hematoxylin difference entropy (*ex vivo*, corr. p=0.0065; *in vivo* corr. p<0.005), mean hematoxylin sum entropy (*ex vivo*, corr. p<0.005; *in vivo* corr. p<0.005), and variance in hematoxylin sum entropy (*ex vivo*, corr. p<0.005; *in vivo* corr. p<0.005).

**Figure 5:**
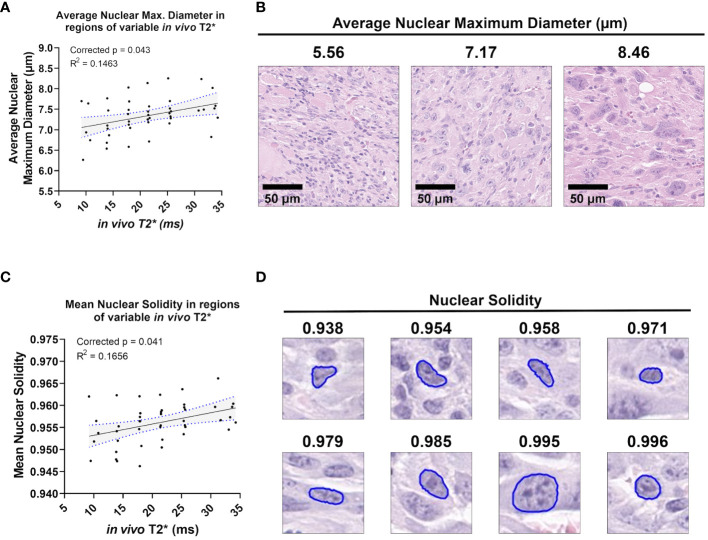
*In vivo* T2* correlates with nuclear topology metrics. Among the cohort of soft tissue sarcomas (n = 8), regions of variable *in vivo* T2* signal demonstrated significant linear relationships with average nuclear maximum diameter **(A)**, a direct measure of nuclear size **(B)**. *In vivo* T2* also correlated significantly with mean nuclear solidity **(C)**. Nuclear solidity is a measure of how much the nuclear boundary deviates from a convex shape, with lower values correlating with greater irregularity **(D)**. Scale bars = 100 µm.

**Figure 6:**
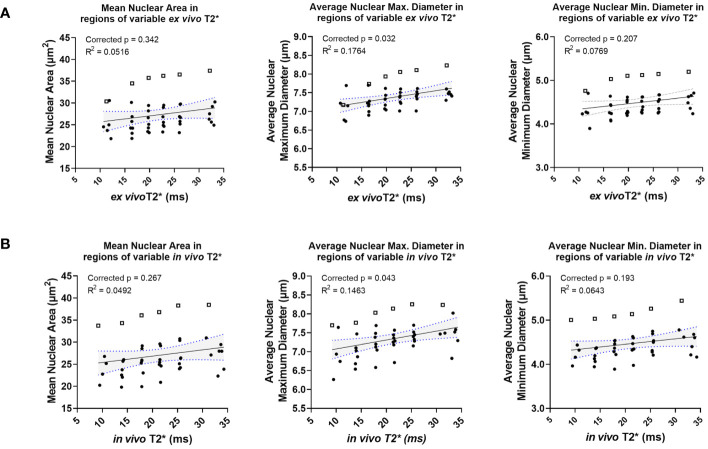
*Ex vivo* and *in vivo* T2* show positive correlations with nuclear size, including in a statistical outlier. *Ex vivo* T2* showed trends of positive correlation with nuclear size metrics, including mean nuclear area, average nuclear maximum diameter, and average nuclear minimum diameter **(A)**. The same trends were seen with *in vivo* T2* and nuclear size metrics **(B)**. In all cases, data from a single tumor (represented in each graph by open squares) exhibited larger nuclear size metrics overall relative to measurements made in all other tumors in the cohort (solid circles).

A small group of nuclear topology features correlated with one or both T2* sequences. *In vivo* T2* showed a positive correlation with nuclear maximum diameter (corr. p=0.0432), a measure of nuclear size (**Figures 5A, B**). This correlation was also seen with *ex vivo* T2* (corr. p=0.0316). Differences in nuclear size are common in human UPS. Also correlating with *in vivo* T2* was mean nuclear solidity (corr. p=0.0412; **Figure 5C**), which measures irregularity of nuclear shape by calculating the difference between the measured area and the convex perimeter (**Figure 5D**). Interestingly, while significant correlations between T2* and nuclear topology features were limited, positive linear trends were noted between both T2* contrasts and measures of nuclear size (**Figure 6**). Along with significant positive linear correlations with nuclear maximum diameter, non-significant positive correlations were also seen with nuclear area (*ex vivo* corr. p=0.3372; *in vivo* corr. p=0.2666) and minimum nuclear diameter (*ex vivo* corr. p=0.2068; *in vivo* corr. p=0.1932). In each case, data from a single tumor diminished the goodness of fit, thus extinguishing statistical significance. For all nuclear size features, data from the same tumor was visibly separable from the rest of the cohort. This tumor showed consistently larger nuclear sizes compared to other mice in the cohort. The source of this difference in nuclear size is unknown. However, the positive correlation between T2* and nuclear size metrics was still observed in this tumor. Taken together, these observations suggest that nuclear size may play a considerable role in the T2* measured in these murine soft tissue sarcomas.

### *In vivo* T2* correlated significantly with variance in nuclear stain intensity features

**Figure 7:**
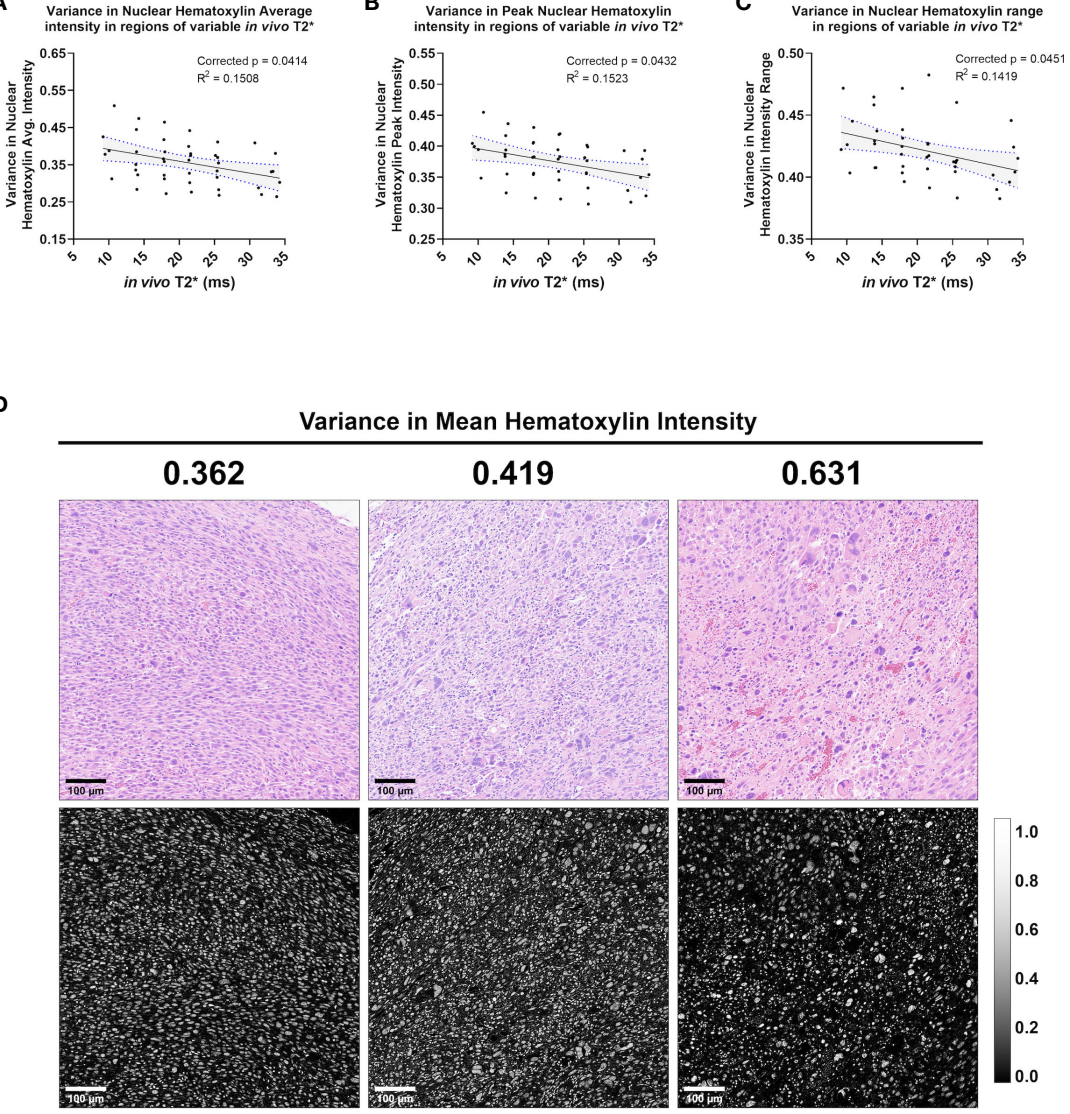
*In vivo* T2* correlates with three measures of variance in nuclear Hematoxylin stain intensity. Among the cohort of soft tissue sarcomas (n = 8), regions of variable *in vivo* T2* signal demonstrated significant linear relationship with variance in nuclear hematoxylin average intensity **(A)**, variance in nuclear hematoxylin peak intensity **(B)**, and variance in nuclear hematoxylin range **(C)**. In each case, variance in hematoxylin intensity metrics showed an inverse correlation with *in vivo* T2*. Regression lines are plotted in black, with 95% CI represented with blue dotted lines and shading. Corrected p-values and R^2^ values are provided for reference. Variance in mean nuclear hematoxylin intensity is a measure of how a cell’s nuclear stain intensity varies from its local neighbors, with high values indicating more heterogeneous groups **(C)**. Shown are representative tiles of H&E-stained soft tissue sarcomas with differing variance in nuclear mean hematoxylin intensity (top) and quantitative hematoxylin stain vector images (bottom) **(D)**. Scale bars = 100 µm.

In addition to nuclear Haralick features and topology metrics, measures of hematoxylin stain intensity also correlated significantly with *in vivo* T2* (**Figure 7**). Specifically, the stain features correlating with *in vivo* T2* were variance metrics, including variance in hematoxylin peak intensity (corr. p=0.0432), variance in hematoxylin average intensity (corr. p=0.0414), and variance in hematoxylin minimum intensity (corr. p=0.0451). Important to note, variance metrics in this case refer to variance among neighboring cells in a 35 μm^2^ pixel. Unlike mean Haralick texture features, which measure intensity patterns within a nucleus, the calculated variance metrics measure differences among neighboring nuclei. In this case, a negative correlation between *in vivo* T2* and hematoxylin variance features suggests that higher T2* may be measured when neighboring cells have similar hematoxylin staining properties (**Figure 7D**).

### *Ex vivo* ADC correlated significantly with three measures of cytometric variance

**Figure 8:**
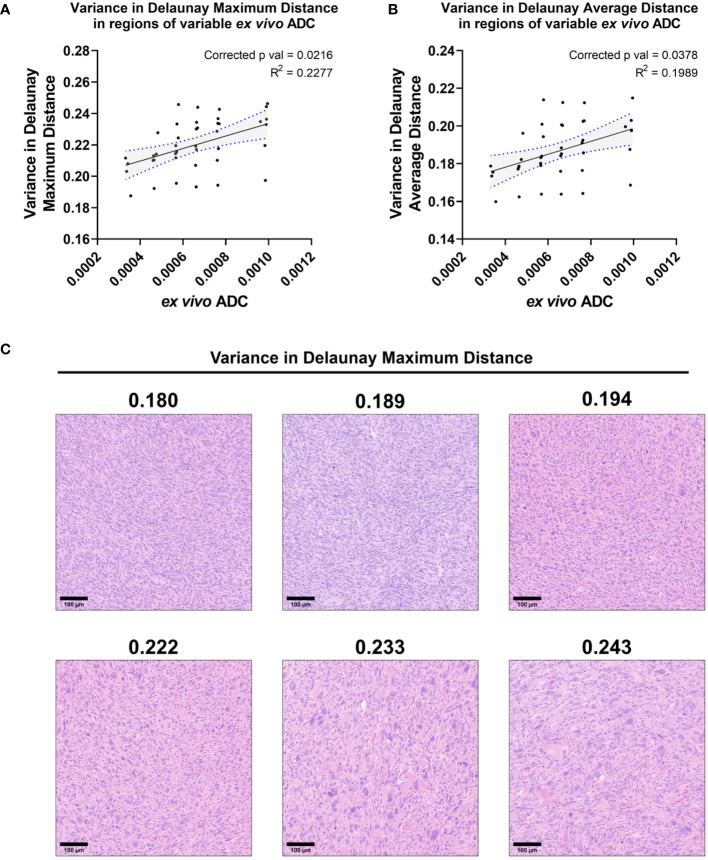
*Ex vivo* ADC correlates with two measures of Delaunay triangulation variance. Among the cohort of soft tissue sarcomas (n=8), regions of variable *ex vivo* ADC demonstrated a significant linear relationship with variance in Delaunay maximum distance **(A)** and Delaunay average distance **(B)**. Regression lines are plotted in black, with 95% CI represented with blue dotted lines and shading. Corrected p-values and R^2^ values are provided for reference. Variance in Delaunay distances measures the heterogeneity of cell-to-cell distances within local populations, demonstrated visually in representative tiles comparing regions of increasing variance of Delaunay maximum distance in soft tissue sarcomas **(C)**. Scale bars = 100 µm.

While numerous cytometric features correlated with T2* in this preclinical sarcoma cohort, very few significant correlations were observed between cytometric features and ADC. No features demonstrated significantly non-zero linear relationships with *in vivo* ADC when corrected for multiple comparisons. When comparing cytometric features to *ex vivo* ADC, three features demonstrated a significant linear relationship: variance in Delaunay maximum distance (corr. p=0.0216), variance in Delaunay average distance (corr. p=0.0378), and variance in nuclear hematoxylin Haralick entropy (corr. p=0.0378). All three features are variance metrics, measuring variance among neighboring cells. Delaunay triangulation measures the distance between a cell and its nearest neighbors. Variance in Delaunay distances, which correlated positively with *ex vivo* ADC, measures the degree to which intercellular distances vary in local cell populations (**Figure 8**). Higher variance in Delaunay distances suggests greater heterogeneity in cell-to-cell distances, often demonstrated in highly pleomorphic regions of these soft tissue sarcomas (**Figure 8C**).

## Discussion

UPS is one of the most aggressive subtypes of sarcoma, but is one of the most poorly categorized. Recent efforts to understand disease behavior have focused on genomic profiling (29), but clinical methods to aid decision making, such as radiological biomarkers, have remained elusive. In response to this deficiency, studies of qMRI parameters, including radiomic features, to predict UPS behavior and response to treatment are being explored with MRI (30). Despite efforts to understand or predict UPS behavior with MR imaging, the histological underpinnings of qMRI in UPS remain unresolved. Clinically, pathologist assessment is still the gold standard for prognostication in UPS, despite the challenge of adequate sampling posed by these heterogeneous and often large tumors. By providing a quantitative link between MR imaging signatures and tissue histology, we can empower the radiologist with histological insights as well as design better, more predictive imaging methods for UPS. To better understand the tissue histology underlying UPS qMRI, we utilized unique imaging libraries in a cohort of autochthonous soft tissue sarcomas in genetically engineered mice that resemble human UPS in both histologic appearance and gene expression. With these rich datasets that include *in vivo* and *ex vivo* MR images registered to tissue pathology slides, we identified significant linear relationships between quantitative MR and tumor cytometric features. In particular, we found a group of cytometric features, including nuclear texture and topology features, which correlated significantly with *in vivo* and *ex vivo* T2*. These studies represent the first of their kind to link qMRI and histological features quantitatively via tissue-registered imaging datasets in a preclinical cohort of UPS.

In this study, the majority of significant linear relationships between cytometric features and qMRI were identified in T2*, both *ex vivo* and *in vivo*. In both cases, nuclear Haralick features were predominant in correlating with T2*. Haralick features are calculated from the gray-level co-occurrence matrix (GLCM) of pixels or voxels within a region of interest (31). Unlike first order image statistics, Haralick features describe the spatial relationships, or “textures”, of normalized grayscale values (32). In the cytometric feature maps, the nuclear Haralick features were measured based on the hematoxylin stain vector, thus describing texture features within individual nuclei. Nuclear Haralick feature quantitation is sensitive to differences between uniformly-stained nuclei and nuclei with heterogeneous or unevenly distributed hematoxylin intensity. An example of this type of distinction would be heterochromatic versus euchromatic nuclei, both of which are seen in UPS (33). We identified linear relationships between T2* and multiple nuclear Haralick features, as well as with local variance in nuclear Haralick features. Nuclear pleomorphism is a hallmark of UPS, thus making T2* a potentially valuable imaging approach for understanding these tumors.

We also found a limited number of nuclear topological features, such as nuclear maximum diameter, which correlated significantly with T2*. Additional nuclear topology features, particularly those related to nuclear size, showed positive trends with T2*. Specifically, increased nuclear sizes were measured in regions with higher T2*. However, many of these linear relationships were not statistically significant due to the presence of single tumor. Although the positive relationships were measured in this tumor as well, the average size of the nuclei measured in this specimen was larger than the rest of the cohort. The cause of this discrepancy is unknown. When considering nuclear size as a possible correlate with T2* in these sarcomas, it is important to note that Haralick features can also be affected by the size of the region in which they are calculated (28, 34). In this case, the Haralick features would be inherently related to nuclear size. Taken together, despite the presence of a single tumor with excessive influence on this dataset, trends between nuclear size metrics and T2* warrant further exploration.

In observing the histology slides, we speculate that the origins of the correlations between cytometric features and T2* arise from differences in cytoarchitecture, size and distribution. Early NMR studies of the relaxation properties of tissues determined that many tissues have multicomponent T1, T2 (35, 36). R_2_^*^, i.e. the rate of decay, is the sum of the rates from all the mechanisms contributing to loss of coherence—molecular, imaging gradients, exchange effects and diffusion. Majumdar and Gore demonstrated that diffusion through susceptibility induced gradients would reduce the transverse relaxation time (37). While our data do not definitively support this mechanism, they are consistent with it. When reflecting on the utility of T2* measurement for studying tumor tissues, it is imperative to consider the relationship between T2* and spatial resolution. Although many of the relationships we found between T2* and histological features were observed in high resolution MRH as well as lower resolution *in vivo* MRI, we have not yet demonstrated the limits of detection of this relationship. Ongoing studies have incorporated these questions into sequence program design and included multi-resolution acquisition protocols. Still, improving our understanding of the pathological insights that T2* mapping may provide to radiologists could impact future clinical care, as nuclear size has been shown to have prognostic significance in multiple sarcomas (38–40). In osteosarcoma, particularly, tumor nuclear size has been shown to correlate with response to chemotherapy (41). In other solid tumors, nuclear size and feature variance, as described above, are associated with tumor grade and aggressiveness (42–44). Although T2* is not widely employed in the clinical setting, further exploration of its utility may add great value to UPS MRI.

While many cytometric features correlated with T2*, we found very few features significantly associated with ADC. This was surprising, given evidence in the literature connecting ADC to tumor pathology, particularly tumor cell density (45–47). Further, the limited correlations noted in *ex vivo* ADC were not seen *in vivo*. In both cases, we believe that deficiencies in the scan protocols may have diminished any findings of interest. Regarding *in vivo* ADC, we hypothesize that the lack of significant relationships may be attributable, in part, to challenges related to signal-to-noise ratio (SNR) with the scan protocol. The bias conferred by the surface coil caused a disproportionate SNR loss in the spiral DWI sequence compared to other sequences, such as the MGRE sequence. Often, this resulted in 30-50% of the tumor volume being irreparably affected by noise, depending on the size or depth of the tumor in the hind limb relative to the surface coil. To address this issue in subsequent studies, we have begun using a modified volume coil and updated the DWI protocol to achieve higher and more uniform SNR.

Results from the *ex vivo* ADC are more challenging to interpret. These images were free of coil-related signal bias and had excellent SNR. In fact, individual tumors demonstrated very strong linear correlations with *ex vivo* ADC, including inverse relationships with features related to cell density as expected based on the literature (48). However, many of these trends were in complete opposing directions for other tumors. As a result, group data for these features showed no significant relationships with *ex vivo* ADC. While challenging to interpret, we hypothesize that these peculiar results were also due to insufficiencies in the scan protocol. Specifically, the *ex vivo* DWI protocol employed multiple *b* values to calculate ADC, but all data were acquired in a single direction, which may be too reductive at such high resolutions. The cells measured within a 35 μm^3^ voxel can demonstrate directional architecture, as these sarcomas frequently include elongated or spindled tumor cells arranged in fascicular or storiform patterns with variable directionality. We hypothesize that at high resolution, the diffusion properties in a single direction may be impacted by this directional tissue structure. Important to note, while the literature generally associates tumor ADC with cell density, there have been discrepancies among studies (49–52). We hypothesize that our data could provide evidence that the DWI sequence parameters may play a role in how ADC relates to cell density. While not used in the present study, we have subsequently employed multi-directional DWI to exploit these features as additional parameters for future tumor analyses.

The primary limitations to this study were deficiencies in scan protocols discussed above and the small sample size for this cohort. Subsequent experiments have overcome many of the inadequacies of the scan protocols based on the confounds identified in this study. While statistically significant associations were observed between cytometric features and qMRI, these results should be interpreted in the context of the limited sample size. Ongoing studies have utilized the data from this pilot study to adjust our statistical models and to calculate suitable, larger cohort sizes. We have also improved our imaging and registration workflows to reduce technical noise in the resulting data. Further, future studies will incorporate more nuanced approaches for comparing cytometric features with qMRI beyond linear relationships, such as non-linear and multivariate analyses. Finally, *in vivo* and *ex vivo* images were acquired on different scanners, which could introduce technical variables when directly comparing *in vivo* and *ex vivo* MR. This has been remedied in subsequent experiments through instrumentation upgrades.

The most notable achievement of this work was demonstrating the ability to consistently measure significant quantitative relationships between soft tissue sarcoma histologic features and qMRI at the intratumoral level in a cohort of animals. These experiments represent the foundation by which tissue histology can directly inform results derived from radiomics studies in UPS (53, 54), thus providing a mechanistic link between qMRI and tumor biology. Our previous work focused primarily on methodological development to facilitate MRI/histology registration and quantitative comparison using MRH. However, in initial experiments we found that inconsistencies in tissue handling and histological preparation on a case-by-case basis, such as poor fixation and inconsistent staining, prevented us from comparing data between tumors. In this study, we employed a series of methodological advancements compared to previous studies to facilitate group data analysis. First, tumor-bearing limbs were perfusion-fixed during euthanasia to improve *ex vivo* tissue quality. Second, the histological technique was improved to ensure maintenance of tissue integrity, as well as to facilitate improved automated histological analysis. Third, we expanded the histological slide analysis to include more features for comparison to MR. Finally, we increased the spatial resolution of the MRH datasets to improve registration to histological slides. With the aforementioned improvements to data collection, these experiments represent the first of their kind to identify statistically significant relationships between cytometric features and qMRI among a cohort of tumor-bearing animals.

With these improvements, we have shown that the heterogeneity of multi-parametric qMRI in sarcomas can be linked to tissue pathology and that pathological hallmarks of UPS, like nuclear pleomorphism, may specifically correlate to select qMRI patterns. Importantly, we achieved image registration from *in vivo* MRI to histology slides by utilizing non-destructive MRH, without the need for any special tissue processing equipment, stereotactic tissue molds, or non-standard histological techniques. The results from this study provide pathology-based insights into qMRI in sarcomas, and can inform thoughtful sequence selection when designing MR imaging biomarker studies in UPS. Further, this work provides a methodological approach for defining the histological basis of tumor radiomics studies. Utilizing cutting-edge imaging techniques and analysis pipelines, these experiments have established quantifiable links between heterogeneous tumor qMRI properties and their histological underpinnings. In this way, we have begun to unravel the specific tissue pathologies which contribute to the radiological presentation of UPS. This work represents an initial key step in empowering the clinical radiologist with pathological insights using non-invasive, whole-tumor MR imaging.

## Data availability statement

The raw data supporting the conclusions of this article will be made available by the authors, without undue reservation.

## Ethics statement

The animal study was approved by Duke University Institutional Animal Care and Use Committee (IACUC). The study was conducted in accordance with the local legislation and institutional requirements.

## Author contributions

SB: Writing – review & editing, Writing – original draft, Visualization, Validation, Methodology, Investigation, Formal analysis, Conceptualization. YM: Writing – review & editing, Writing – original draft, Visualization, Supervision, Resources, Project administration, Investigation, Data curation, Conceptualization. JE: Writing – review & editing, Validation, Supervision, Methodology, Conceptualization. JC: Writing – review & editing, Software, Methodology. GC: Writing – review & editing, Investigation, Data curation. YQ: Writing – review & editing, Investigation, Data curation. AB: Writing – review & editing, Investigation, Data curation. EX: Writing – review & editing, Investigation, Data curation. DK: Writing – review & editing, Supervision, Resources, Project administration, Methodology, Funding acquisition. CB: Writing – review & editing, Supervision, Resources, Project administration, Methodology, Funding acquisition. GJ: Writing – review & editing, Supervision, Resources, Project administration, Methodology, Funding acquisition.
