# Supplementary material for: MR histology reveals tissue features beneath heterogeneous MRI signal in genetically engineered mouse models of sarcoma
Source: Front Oncol. 2024 May 31;14:1287479. doi: 10.3389/fonc.2024.1287479 (PMC11176416; doi:10.3389/fonc.2024.1287479)
Supplement: Supplementary file 1 [file Table_1.docx]

Supplementary Material

# Supplementary Table 1

| **Supplementary Table 1. Image registration metrics describing *in vivo* and *ex vivo* MR registration to histology slides for all animals on study.** | | | | | | |
| --- | --- | --- | --- | --- | --- | --- |
| **Specimen** | **Image** | **Dice Score** | **% False Positives** | **% False Negatives** | **Average HD (µm)** | **95% Max HD (µm)** |
| 1 | *Ex vivo* | 0.947 | 2.69 | 3.92 | 7.6 | 42.8 |
|  | *In vivo* | 0.946 | 4.84 | 1.80 | 5.9 | 26.3 |
| 2 | *Ex vivo* | 0.937 | 3.07 | 4.14 | 5.8 | 35.0 |
|  | *In vivo* | 0.932 | 4.35 | 3.23 | 6.9 | 52.5 |
| 3 | *Ex vivo* | 0.958 | 2.39 | 2.54 | 4.8 | 8.8 |
|  | *In vivo* | 0.912 | 5.07 | 5.19 | 16.7 | 115.8 |
| 4 | *Ex vivo* | 0.958 | 1.43 | 3.77 | 4.7 | 19.3 |
|  | *In vivo* | 0.949 | 3.47 | 2.58 | 6.2 | 21.9 |
| 5 | *Ex vivo* | 0.944 | 3.30 | 4.15 | 5.6 | 17.5 |
|  | *In vivo* | 0.934 | 4.79 | 3.53 | 6.8 | 42.2 |
| 6 | *Ex vivo* | 0.970 | 1.35 | 2.37 | 2.1 | >5.0 |
|  | *In vivo* | 0.964 | 2.48 | 2.01 | 2.3 | >0.5 |
| 7 | *Ex vivo* | 0.917 | 2.99 | 5.60 | 7.7 | 56.6 |
|  | *In vivo* | 0.909 | 5.70 | 3.70 | 11.5 | 77.2 |
| 8 | *Ex vivo* | 0.922 | 3.47 | 1.60 | 11.7 | 56.6 |
|  | *In vivo* | 0.891 | 5.07 | 2.20 | 14.8 | 87.8 |
| **GROUP** | ***Ex vivo*** | **0.944** | **2.59** | **3.51** | **6.2** | **29.6** |
|  | ***In vivo*** | **0.930** | **4.47** | **3.03** | **8.9** | **53.0** |
