# Supplementary material for: MR histology reveals tissue features beneath heterogeneous MRI signal in genetically engineered mouse models of sarcoma
Source: Front Oncol. 2024 May 31;14:1287479. doi: 10.3389/fonc.2024.1287479 (PMC11176416; doi:10.3389/fonc.2024.1287479)
Supplement: Supplementary file 2 [file Table_2.docx]

Supplementary Material

# Supplementary Table 2

| **Supplemental Table 2. Significance of non-zero linear relationships between intra-tumoral *ex vivo* ADC and cytometric features in soft tissue sarcomas (n = 8) corrected for multiple comparisons** | | | |
| --- | --- | --- | --- |
| **Category** | **Feature** | **p-value** | **Benjamini-Hochberg** |
|  |  |  | **corrected p-value** |
| Topology | Detection Count | 0.2118 | 0.3689 |
|  | Mean Nuclear Diameter Ratio | 0.0590 | 0.1593 |
|  | Variance in Nuclear Diameter Ratio | 0.1472 | 0.2944 |
|  | Mean Nuclear Area | 0.5489 | 0.6444 |
|  | Variance in Nuclear Area | 0.0351 | 0.1264 |
|  | Mean Nuclear Circularity | 0.3107 | 0.4661 |
|  | Variance in Nuclear Circularity | 0.3537 | 0.4897 |
|  | Mean Nuclear Maximum Diameter | 0.3560 | 0.4806 |
|  | Variance in Nuclear Maximum Diameter | 0.0823 | 0.2020 |
|  | Mean Nuclear Minimum Diameter | 0.5947 | 0.6833 |
|  | Variance in Nuclear Minimum Diameter | 0.0403 | 0.1360 |
|  | Mean Nuclear Solidity | 0.7016 | 0.7732 |
|  | Variance in Nuclear Solidity | 0.2960 | 0.4567 |
| Delaunay | Mean Delaunay Maximum Distance | 0.1064 | 0.2498 |
|  | Variance in Delaunay Maximum Distance | 0.0016 | **0.0216** |
|  | Mean Delaunay Average Distance | 0.1493 | 0.2879 |
|  | Variance in Delaunay Average Distance | 0.0035 | **0.0378** |
|  | Mean Delaunay Minimum Distance | 0.2886 | 0.4723 |
|  | Variance in Delaunay Minimum Distance | 0.0175 | 0.1050 |
|  | Mean Delaunay Ratio | 0.0289 | 0.1200 |
|  | Variance in Delaunay Ratio | 0.0142 | 0.0959 |
|  | Mean Delaunay Triangle Area | 0.1188 | 0.2673 |
|  | Variance in Delaunay Triangle Area | 0.0197 | 0.0967 |
|  | Mean Delaunay Number of Neighbors | 0.1229 | 0.2655 |
|  | Variance in Delaunay Number of Neighbors | 0.0404 | 0.1283 |
| Nuclear Haralick | Mean Hematoxylin ASM | 0.0066 | 0.0594 |
|  | Variance in Hematoxylin ASM | 0.3790 | 0.4992 |
|  | Mean Hematoxylin Contrast | 0.2438 | 0.4114 |
|  | Variance in Hematoxylin Contrast | 0.8181 | 0.8662 |
|  | Mean Hematoxylin Correlation | 0.1706 | 0.3071 |
|  | Mean Hematoxylin Difference Entropy | 0.4990 | 0.5988 |
|  | Variance Hematoxylin Difference Entropy | 0.0014 | **0.0378** |
|  | Mean Hematoxylin Entropy | 0.1556 | 0.2897 |
|  | Variance Hematoxylin Entropy | 0.0014 | 0.0252 |
|  | Mean Hematoxylin Inverse Difference Moment | 0.8296 | 0.8615 |
|  | Variance in Hematoxylin Inverse Difference Moment | 0.0558 | 0.1586 |
|  | Mean Hematoxylin IMC1 | 0.8484 | 0.8484 |
|  | Variance in Hematoxylin IMC1 | 0.4031 | 0.5183 |
|  | Mean Hematoxylin IMC2 | 0.1258 | 0.2613 |
|  | Variance in Hematoxylin IMC2 | 0.0480 | 0.1440 |
|  | Mean Hematoxylin Sum of Squares | 0.4181 | 0.5131 |
|  | Variance in Hematoxylin Sum of Squares | 0.3508 | 0.4985 |
|  | Mean Hematoxylin Sum Average | 0.8018 | 0.8659 |
|  | Variance in Hematoxylin Sum Average | 0.0177 | 0.0956 |
|  | Mean Hematoxylin Sum Entropy | 0.0668 | 0.1718 |
|  | Variance Hematoxylin Sum Entropy | 0.0011 | 0.0594 |
| Stain | Mean Hematoxylin Peak Intensity | 0.8423 | 0.8582 |
|  | Variance in Hematoxylin Peak Intensity | 0.0229 | 0.1031 |
|  | Mean Hematoxylin Average Intensity | 0.6909 | 0.7773 |
|  | Variance in Hematoxylin Average Intensity | 0.0125 | 0.0964 |
|  | Mean Hematoxylin Range | 0.4102 | 0.5151 |
|  | Variance in Hematoxylin Range | 0.0329 | 0.1269 |
|  | Mean Hematoxylin Standard Deviation | 0.3361 | 0.4905 |
|  | Variance in Hematoxylin Standard Deviation | 0.2929 | 0.4652 |
| *Statistically significant p-values (corrected p < 0.05) are shown in bold and highlighted blue.*  *ADC, apparent diffusion coefficient; ASM, angular second moment; IMC, informational measure of correlation.* | | | |
