# Supplementary material for: MR histology reveals tissue features beneath heterogeneous MRI signal in genetically engineered mouse models of sarcoma
Source: Front Oncol. 2024 May 31;14:1287479. doi: 10.3389/fonc.2024.1287479 (PMC11176416; doi:10.3389/fonc.2024.1287479)
Supplement: Supplementary file 3 [file Table_3.docx]

Supplementary Material

# Supplementary Table 3

| **Supplemental Table 3. Significance of non-zero linear relationships between intra-tumoral *in vivo* ADC and cytometric features in soft tissue sarcomas (n = 8) corrected for multiple comparisons** | | | |
| --- | --- | --- | --- |
| **Category** | **Feature** | **p-value** | **Benjamini-Hochberg** |
|  |  |  | **corrected p-value** |
| Topology | Detection Count | 0.4161 | 0.9362 |
|  | Mean Nuclear Diameter Ratio | 0.7675 | 0.8458 |
|  | Variance in Nuclear Diameter Ratio | 0.6737 | 0.7579 |
|  | Mean Nuclear Area | 0.6049 | 0.7777 |
|  | Variance in Nuclear Area | 0.3896 | 1.0018 |
|  | Mean Nuclear Circularity | 0.5999 | 0.7901 |
|  | Variance in Nuclear Circularity | 0.5292 | 0.9854 |
|  | Mean Nuclear Maximum Diameter | 0.6370 | 0.7644 |
|  | Variance in Nuclear Maximum Diameter | 0.4033 | 0.9469 |
|  | Mean Nuclear Minimum Diameter | 0.6289 | 0.7898 |
|  | Variance in Nuclear Minimum Diameter | 0.4760 | 0.9520 |
|  | Mean Nuclear Solidity | 0.6735 | 0.7738 |
|  | Variance in Nuclear Solidity | 0.1335 | 0.5545 |
| Delaunay | Mean Delaunay Maximum Distance | 0.5726 | 0.8589 |
|  | Variance in Delaunay Maximum Distance | 0.5365 | 0.9053 |
|  | Mean Delaunay Average Distance | 0.5743 | 0.8382 |
|  | Variance in Delaunay Average Distance | 0.5349 | 0.9318 |
|  | Mean Delaunay Minimum Distance | 0.5845 | 0.8093 |
|  | Variance in Delaunay Minimum Distance | 0.5653 | 0.8722 |
|  | Mean Delaunay Ratio | 0.5165 | 0.9961 |
|  | Variance in Delaunay Ratio | 0.5433 | 0.8890 |
|  | Mean Delaunay Triangle Area | 0.6419 | 0.7535 |
|  | Variance in Delaunay Triangle Area | 0.5324 | 0.9583 |
|  | Mean Delaunay Number of Neighbors | 0.8352 | 0.8673 |
|  | Variance in Delaunay Number of Neighbors | 0.4441 | 0.9224 |
| Nuclear Haralick | Mean Hematoxylin ASM | 0.3963 | 0.9727 |
|  | Variance in Hematoxylin ASM | 0.2427 | 0.7709 |
|  | Mean Hematoxylin Contrast | 0.0978 | 0.5868 |
|  | Variance in Hematoxylin Contrast | 0.3275 | 0.8843 |
|  | Mean Hematoxylin Correlation | 0.0192 | 1.0368 |
|  | Mean Hematoxylin Difference Entropy | 0.8159 | 0.8639 |
|  | Variance Hematoxylin Difference Entropy | 0.8523 | 0.8684 |
|  | Mean Hematoxylin Entropy | 0.5903 | 0.7969 |
|  | Variance Hematoxylin Entropy | 0.5488 | 0.8716 |
|  | Mean Hematoxylin Inverse Difference Moment | 0.0729 | 0.5624 |
|  | Variance in Hematoxylin Inverse Difference Moment | 0.2059 | 0.6949 |
|  | Mean Hematoxylin IMC1 | 0.0763 | 0.5150 |
|  | Variance in Hematoxylin IMC1 | 0.1179 | 0.5788 |
|  | Mean Hematoxylin IMC2 | 0.1157 | 0.6248 |
|  | Variance in Hematoxylin IMC2 | 0.3260 | 0.9265 |
|  | Mean Hematoxylin Sum of Squares | 0.0280 | 0.3024 |
|  | Variance in Hematoxylin Sum of Squares | 0.2573 | 0.7719 |
|  | Mean Hematoxylin Sum Average | 0.0226 | 0.6102 |
|  | Variance in Hematoxylin Sum Average | 0.6340 | 0.7781 |
|  | Mean Hematoxylin Sum Entropy | 0.1424 | 0.5493 |
|  | Variance Hematoxylin Sum Entropy | 0.5773 | 0.8204 |
| Stain | Mean Hematoxylin Peak Intensity | 0.0251 | 0.4518 |
|  | Variance in Hematoxylin Peak Intensity | 0.9145 | 0.9145 |
|  | Mean Hematoxylin Average Intensity | 0.0273 | 0.3686 |
|  | Variance in Hematoxylin Average Intensity | 0.7854 | 0.8482 |
|  | Mean Hematoxylin Range | 0.1214 | 0.5463 |
|  | Variance in Hematoxylin Range | 0.4398 | 0.9500 |
|  | Mean Hematoxylin Standard Deviation | 0.0554 | 0.4986 |
|  | Variance in Hematoxylin Standard Deviation | 0.1710 | 0.6156 |
| *ADC, apparent diffusion coefficient; ASM, angular second moment; IMC, informational measure of correlation.* | | | |
