# Supplementary material for: MR histology reveals tissue features beneath heterogeneous MRI signal in genetically engineered mouse models of sarcoma
Source: Front Oncol. 2024 May 31;14:1287479. doi: 10.3389/fonc.2024.1287479 (PMC11176416; doi:10.3389/fonc.2024.1287479)
Supplement: Supplementary file 4 [file Table_4.docx]

Supplementary Material

# Supplementary Table 4

| **Supplemental Table 4. Significance of non-zero linear relationships between intra-tumoral *ex vivo* T2* and cytometric features in soft tissue sarcomas (n = 8) corrected for multiple comparisons** | | | |
| --- | --- | --- | --- |
| **Category** | **Feature** | **p-value** | **Benjamini-Hochberg** |
|  |  |  | **corrected p-value** |
| Topology | Detection Count | 0.8192 | 0.8847 |
|  | Mean Nuclear Diameter Ratio | 0.9816 | 0.9816 |
|  | Variance in Nuclear Diameter Ratio | 0.8063 | 0.9264 |
|  | Mean Nuclear Area | 0.1333 | 0.3372 |
|  | Variance in Nuclear Area | 0.5952 | 0.8035 |
|  | Mean Nuclear Circularity | 0.2466 | 0.4932 |
|  | Variance in Nuclear Circularity | 0.4785 | 0.7383 |
|  | Mean Nuclear Maximum Diameter | 0.0041 | **0.0316** |
|  | Variance in Nuclear Maximum Diameter | 0.6342 | 0.8154 |
|  | Mean Nuclear Minimum Diameter | 0.0651 | 0.2068 |
|  | Variance in Nuclear Minimum Diameter | 0.3800 | 0.7076 |
|  | Mean Nuclear Solidity | 0.0100 | 0.0675 |
|  | Variance in Nuclear Solidity | 0.1925 | 0.4158 |
| Delaunay | Mean Delaunay Maximum Distance | 0.8091 | 0.8917 |
|  | Variance in Delaunay Maximum Distance | 0.0187 | 0.0918 |
|  | Mean Delaunay Average Distance | 0.9756 | 0.9940 |
|  | Variance in Delaunay Average Distance | 0.0486 | 0.1750 |
|  | Mean Delaunay Minimum Distance | 0.4760 | 0.7560 |
|  | Variance in Delaunay Minimum Distance | 0.1133 | 0.2913 |
|  | Mean Delaunay Ratio | 0.0950 | 0.2700 |
|  | Variance in Delaunay Ratio | 0.0431 | 0.1662 |
|  | Mean Delaunay Triangle Area | 0.9370 | 0.9730 |
|  | Variance in Delaunay Triangle Area | 0.0350 | 0.1454 |
|  | Mean Delaunay Number of Neighbors | 0.4545 | 0.7917 |
|  | Variance in Delaunay Number of Neighbors | 0.1414 | 0.3320 |
| Nuclear Haralick | Mean Hematoxylin ASM | <0.0001 | **<0.005** |
|  | Variance in Hematoxylin ASM | 0.5456 | 0.7753 |
|  | Mean Hematoxylin Contrast | 0.4607 | 0.7539 |
|  | Variance in Hematoxylin Contrast | 0.8382 | 0.8875 |
|  | Mean Hematoxylin Correlation | 0.2377 | 0.4937 |
|  | Mean Hematoxylin Difference Entropy | <0.0001 | **<0.005** |
|  | Variance Hematoxylin Difference Entropy | 0.0006 | **0.0065** |
|  | Mean Hematoxylin Entropy | <0.0001 | **<0.005** |
|  | Variance Hematoxylin Entropy | 0.0162 | 0.0875 |
|  | Mean Hematoxylin Inverse Difference Moment | 0.8088 | 0.9099 |
|  | Variance in Hematoxylin Inverse Difference Moment | 0.1085 | 0.2930 |
|  | Mean Hematoxylin IMC1 | 0.2632 | 0.5076 |
|  | Variance in Hematoxylin IMC1 | 0.7428 | 0.8720 |
|  | Mean Hematoxylin IMC2 | 0.6852 | 0.8409 |
|  | Variance in Hematoxylin IMC2 | 0.1510 | 0.3398 |
|  | Mean Hematoxylin Sum of Squares | 0.6244 | 0.8224 |
|  | Variance in Hematoxylin Sum of Squares | 0.5515 | 0.7636 |
|  | Mean Hematoxylin Sum Average | 0.5373 | 0.7842 |
|  | Variance in Hematoxylin Sum Average | 0.0750 | 0.2250 |
|  | Mean Hematoxylin Sum Entropy | <0.0001 | **<0.005** |
|  | Variance Hematoxylin Sum Entropy | 0.0012 | **0.0108** |
| Stain | Mean Hematoxylin Peak Intensity | 0.4526 | 0.8147 |
|  | Variance in Hematoxylin Peak Intensity | 0.0270 | 0.1215 |
|  | Mean Hematoxylin Average Intensity | 0.4585 | 0.7737 |
|  | Variance in Hematoxylin Average Intensity | 0.0543 | 0.1833 |
|  | Mean Hematoxylin Range | 0.6645 | 0.8345 |
|  | Variance in Hematoxylin Range | 0.0114 | 0.0684 |
|  | Mean Hematoxylin Standard Deviation | 0.7258 | 0.8710 |
|  | Variance in Hematoxylin Standard Deviation | 0.4935 | 0.7403 |
| *Statistically significant p-values (corrected p < 0.05) are shown in bold and highlighted blue.*  *ASM, angular second moment; IMC, informational measure of correlation.* | | | |
