# Supplementary material for: MR histology reveals tissue features beneath heterogeneous MRI signal in genetically engineered mouse models of sarcoma
Source: Front Oncol. 2024 May 31;14:1287479. doi: 10.3389/fonc.2024.1287479 (PMC11176416; doi:10.3389/fonc.2024.1287479)
Supplement: Supplementary file 5 [file Table_5.docx]

Supplementary Material

# Supplementary Table 5

| **Supplemental Table 5. Significance of non-zero linear relationships between intra-tumoral *in vivo* T2* and cytometric features in soft tissue sarcomas (N=8) corrected for multiple comparisons** | | | |
| --- | --- | --- | --- |
| **Category** | **Feature** | **p-value** | **Benjamini-Hochberg** |
|  |  |  | **corrected p-value** |
| Topology | Detection Count | 0.6707 | 0.7391 |
|  | Mean Nuclear Diameter Ratio | 0.5338 | 0.6551 |
|  | Variance in Nuclear Diameter Ratio | 0.8302 | 0.8790 |
|  | Mean Nuclear Area | 0.1481 | 0.2666 |
|  | Variance in Nuclear Area | 0.3999 | 0.5683 |
|  | Mean Nuclear Circularity | 0.2844 | 0.4388 |
|  | Variance in Nuclear Circularity | 0.6180 | 0.7255 |
|  | Mean Nuclear Maximum Diameter | 0.0104 | **0.0432** |
|  | Variance in Nuclear Maximum Diameter | 0.4563 | 0.5867 |
|  | Mean Nuclear Minimum Diameter | 0.0966 | 0.1932 |
|  | Variance in Nuclear Minimum Diameter | 0.5322 | 0.6683 |
|  | Mean Nuclear Solidity | 0.0061 | **0.0412** |
|  | Variance in Nuclear Solidity | 0.0601 | 0.1545 |
| Delaunay | Mean Delaunay Maximum Distance | 0.9512 | 0.9691 |
|  | Variance in Delaunay Maximum Distance | 0.0077 | **0.0462** |
|  | Mean Delaunay Average Distance | 0.2101 | 0.3438 |
|  | Variance in Delaunay Average Distance | 0.0453 | 0.1223 |
|  | Mean Delaunay Minimum Distance | 0.3867 | 0.5644 |
|  | Variance in Delaunay Minimum Distance | 0.0762 | 0.1870 |
|  | Mean Delaunay Ratio | 0.0934 | 0.1940 |
|  | Variance in Delaunay Ratio | 0.0375 | 0.1066 |
|  | Mean Delaunay Triangle Area | 0.9243 | 0.9599 |
|  | Variance in Delaunay Triangle Area | 0.0189 | 0.0638 |
|  | Mean Delaunay Number of Neighbors | 0.4391 | 0.5928 |
|  | Variance in Delaunay Number of Neighbors | 0.1350 | 0.2514 |
| Nuclear Haralick | Mean Hematoxylin ASM | <0.0001 | **<0.005** |
|  | Variance in Hematoxylin ASM | 0.6720 | 0.7258 |
|  | Mean Hematoxylin Contrast | 0.0923 | 0.1994 |
|  | Variance in Hematoxylin Contrast | 0.0816 | 0.1916 |
|  | Mean Hematoxylin Correlation | 0.0370 | 0.1110 |
|  | Mean Hematoxylin Difference Entropy | 0.0026 | **0.0201** |
|  | Variance Hematoxylin Difference Entropy | <0.0001 | **<0.005** |
|  | Mean Hematoxylin Entropy | 0.0002 | **<0.005** |
|  | Variance Hematoxylin Entropy | 0.0022 | **0.0198** |
|  | Mean Hematoxylin Inverse Difference Moment | 0.4329 | 0.5994 |
|  | Variance in Hematoxylin Inverse Difference Moment | 0.0077 | **0.0416** |
|  | Mean Hematoxylin IMC1 | 0.4549 | 0.5991 |
|  | Variance in Hematoxylin IMC1 | 0.6636 | 0.7466 |
|  | Mean Hematoxylin IMC2 | 0.2089 | 0.3525 |
|  | Variance in Hematoxylin IMC2 | 0.1105 | 0.2131 |
|  | Mean Hematoxylin Sum of Squares | 0.2165 | 0.3439 |
|  | Variance in Hematoxylin Sum of Squares | 0.0358 | 0.1137 |
|  | Mean Hematoxylin Sum Average | 0.9927 | 0.9927 |
|  | Variance in Hematoxylin Sum Average | 0.0131 | **0.0472** |
|  | Mean Hematoxylin Sum Entropy | <0.0001 | **<0.005** |
|  | Variance Hematoxylin Sum Entropy | 0.0001 | **<0.005** |
| Stain | Mean Hematoxylin Peak Intensity | 0.3140 | 0.4710 |
|  | Variance in Hematoxylin Peak Intensity | 0.0088 | **0.0432** |
|  | Mean Hematoxylin Average Intensity | 0.6443 | 0.7403 |
|  | Variance in Hematoxylin Average Intensity | 0.0092 | **0.0414** |
|  | Mean Hematoxylin Range | 0.5567 | 0.6680 |
|  | Variance in Hematoxylin Range | 0.0117 | **0.0451** |
|  | Mean Hematoxylin Standard Deviation | 0.1869 | 0.3256 |
|  | Variance in Hematoxylin Standard Deviation | 0.0870 | 0.1958 |
| *Statistically significant p-values (corrected p < 0.05) are shown in bold and highlighted blue.*  *ASM, angular second moment; IMC, informational measure of correlation.* | | | |
